# Species’ urbanization time but not present urban tolerance predicts avian fear responses towards human

**DOI:** 10.1186/s12915-025-02427-0

**Published:** 2025-10-02

**Authors:** Peter Mikula, Jan Grünwald, Jiří Reif

**Affiliations:** 1 TUM School of Life Sciences, Ecoclimatology, Technical University of Munich, Hans-Carl-von-Carlowitz-Platz 2, Freising, 85354 Germany; 2 Institute for Advanced Study, Technical University of Munich, Lichtenbergstraße 2a, Garching, 85748 Germany; 3 Faculty of Environmental Sciences, Czech University of Life Sciences Prague, Kamýcká 129, Prague, 16500 Czech Republic; 4 Institute for Environmental Studies, Faculty of Science, Charles University, Benátská 2, Prague, 12801 Czechia; 5 Department of Zoology, Faculty of Science, Palacký University, 17. Listopadu 50, Olomouc, 77146 Czechia

**Keywords:** Birds, Flight initiation distance, Monitoring, Urban habitats, Urban tolerance, Urbanization time

## Abstract

**Background:**

Urban environments exert strong pressures on animal behavior, leading to altered fear responses to humans. Species with a longer history of urban presence and greater tolerance to urban environments are expected to show reduced fear responses towards humans. Here, we examined whether avian flight initiation distance (a proxy of fear)—the distance at which a bird flees from an approaching human—is associated with a species’ timing of urban colonization (i.e., when it has started to breed in urban areas) and with present-day urban tolerance (i.e., how common it is in the city). Unlike previous studies which paired avian fear responses and urbanization timing from different regions, we collected both in the same city (Prague, Czechia), minimizing regional differences in urban history and providing a more rigorous test of the link between urbanization timing and avian fear responses.

**Results:**

Using standardized data from 4420 flight initiation distance observations across 68 species, we applied Bayesian phylogenetic mixed models while controlling for ecological and contextual variables. We found that species with a longer urban history (i.e., earlier timing of urban colonization) showed significantly shorter flight initiation distances, suggesting reduced fear responses. In contrast, present-day urban tolerance based on breeding commonness was not related to flight initiation distance variation.

**Conclusions:**

We found that the timing of urban colonization better predicts reduced fear of humans in birds than present-day urban tolerance, emphasizing the role of long-term behavioral filtering and/or selection in shaping urban wildlife behavior. By explicitly separating urbanization time from contemporary urban commonness within a single city and analyzing individual-level fear responses, our study shows that earlier urban colonizers exhibit consistently shorter escape distances, reflecting cumulative long-term processes rather than short-term plasticity alone. These findings highlight the importance of incorporating urban colonization history into behavioral ecology and urban wildlife management frameworks.

**Supplementary Information:**

The online version contains supplementary material available at 10.1186/s12915-025-02427-0.

## Background

Urban development is a rapidly expanding global human-driven process that profoundly alters environmental conditions [1, 2]. As natural landscapes are replaced with anthropogenic environments, many species and populations are forced to either adjust their ecology to novel urban conditions or retreat from them. The colonization of urban environments (hereafter “urbanization”) by animals often coincides with major shifts in their behavior, physiology, and life-history traits [3–6]. One key behavioral adaptation is a reduced fear response to humans, commonly measured as flight initiation distance (FID)—the distance at which an individual animal flees from an approaching person [7, 8]. Urban animals generally exhibit shorter escape distances compared to their rural conspecifics, a phenomenon that is usually interpreted as reflecting their increased tolerance of human proximity and their decreased fear response towards humans [3, 9, 10].

Such shifts in boldness towards humans likely reflect broader shifts in animals’ behavioral syndromes—i.e., consistent sets of correlated behaviors in the same individual expressed across different contexts [11]—as urbanization process may simultaneously select for bold, aggressive, and explorative individuals, yet these traits may vary independently [12–14]. Reduction in animal responsiveness towards human stimulus is often interpreted as the result of habituation, where repeated exposure to benign stimuli (in this case an urban human) leads to diminished responsiveness [15–17]. An alternative explanation is that urban colonization is shaped by pre-existing behavioral traits in ancestral rural populations, such as low wariness or boldness, followed by local selection or non-random settlement of tolerant individuals [4, 18–21]. Consequently, species that have been present in cities for longer periods may exhibit greater tolerance due to sustained exposure to urban environments and repeated filtering/selection for boldness [22, 23].

Time since urbanization is particularly relevant because it reflects not just how long a species has been exposed to anthropogenic environments, but also the evolutionary and ecological processes that may unfold during prolonged urban residence [24–26]. Early colonizers likely underwent strong behavioral filtering, where only individuals with specific traits—such as low flight initiation distance and/or high behavioral flexibility—were able to persist and reproduce in the novel, human-dominated context [22, 27]. Over time, these populations may have become more behaviorally homogenous, with reduced variation in escape responses [22]. Furthermore, long-term urbanized species may face fewer costs for boldness due to lower predation risk in some cities, allowing reduced antipredator behavior to persist or intensify across generations [28]. Understanding how this historical dimension of urban life influences fear responses of wildlife helps us better understand whether urban tolerance reflects plastic, short-term adjustment or deeper, trait-based processes.

Urban colonization is often biased towards species that already show elevated tolerance to human presence, such as reduced fear responses, high boldness, or behavioral flexibility [4, 22]. These pre-adapted traits may facilitate initial penetration and persistence in anthropogenic habitats, suggesting that present urban tolerance—commonness of animals in urban areas—may result from environmental filtering on personality traits and structures [18, 20]. Under this interpretation, species that are more widespread in urban areas today are expected to show reduced fear of humans, as they may possess traits that predispose them to urban life. However, because urban tolerance captures current distribution patterns rather than historical exposure (although current distribution may reflect historical exposure), it may miss longer-term dynamics that shape behavioral adaptation. If urban tolerance plays a key role, we would expect that species more common in urban habitats also exhibit consistently shorter flight initiation distances.

In this study, we test the effects of timing of urban colonization (hereafter, “urbanization time”) and urban tolerance on avian escape behavior, measured as a flight initiation distance, in a metropolitan area. Birds are among the most visible and widely studied urban wildlife, and their responses to human disturbance offer key insights into behavioral adaptation in cities [14, 27]. Using standardized escape distance measurements across multiple bird species in Prague, Czechia, we tested whether species with longer urban history (i.e., urbanization time) show reduced fear of humans and whether this is independent of how common and widespread they are in the urban landscape nowadays (i.e., urban tolerance) while controlling for potentially confounding effects of several contextual and environmental variables. Unlike previous studies which paired avian fear responses and urbanization timing from different regions [22, 23], we collected both from the same city (Prague), minimizing regional differences in urban history and providing a more rigorous test of the link between urbanization timing and avian fear responses. Moreover, by analyzing individual-level fear responses rather than species-averaged values as did in [22, 23], we retained within-species variation, enabling a more precise assessment of how urbanization timing predicts species-specific behavioral tolerance of birds to humans.

## Results

**Fig. 1:**
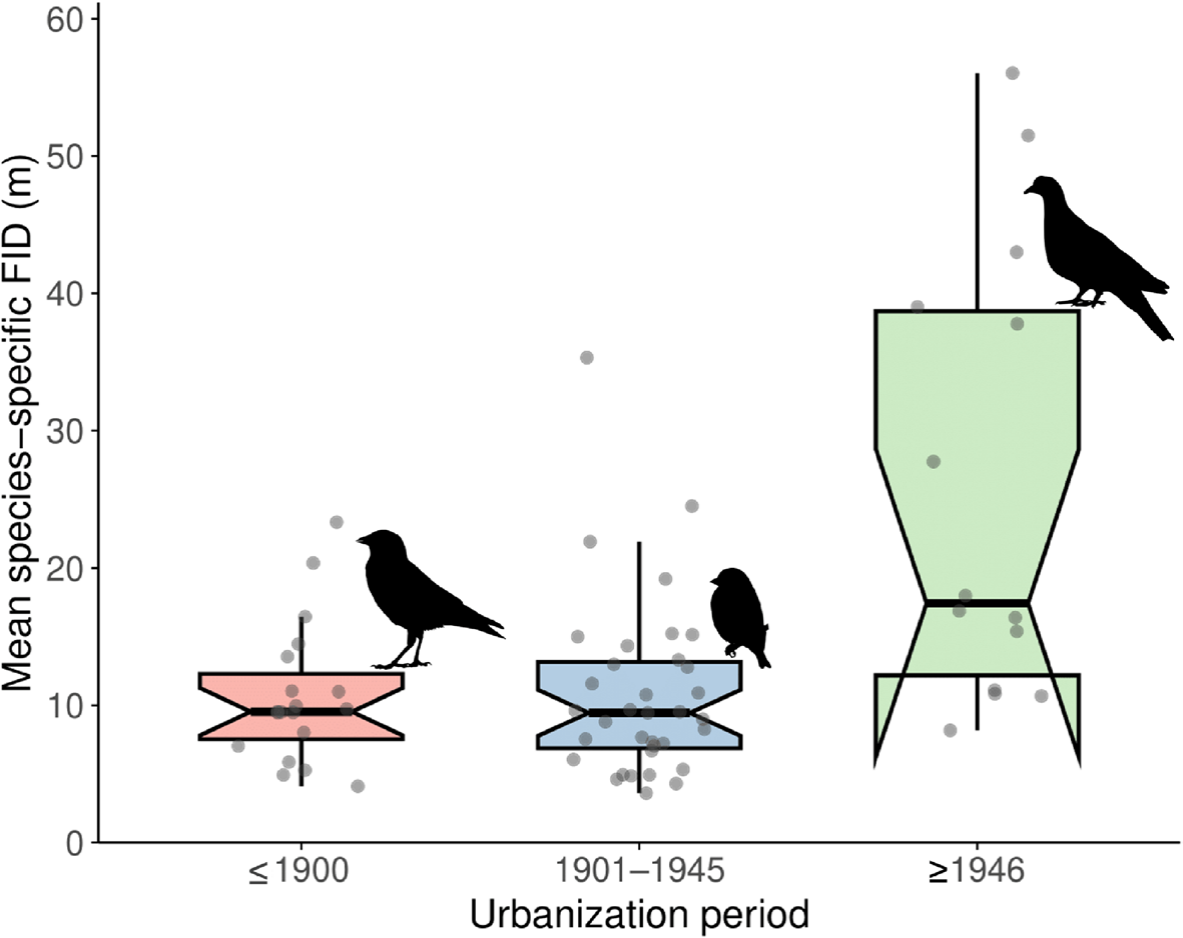
Boxplot showing raw data of species-level mean flight initiation distance (FID) (*N* = 68 species) grouped by the historical period in which each species was first recorded breeding in urban area of Prague city, Czechia. Categories are defined as ≤ 1900, 1901–1945, and 1946–present. Each box represents the interquartile range (IQR), with the horizontal line indicating the median. Whiskers extend to 1.5 times the IQR, and mean species-specific FID values are overlaid as jittered points to illustrate the data distribution. Notches represent approximate 95% confidence intervals around the median; non-overlapping notches suggest a statistically significant difference between groups (irregular notch shape in ≥ 1946 arises because the CI exceeds the box due to large IQR/√*N*). Bird silhouettes represent species that started their urbanization in Prague during the corresponding historical periods (from left to right): western jackdaw (*Coloeus monedula*), European greenfinch (*Chloris chloris*), and common wood pigeon (*Columba palumbus*). Silhouettes were downloaded from PhyloPic (http://phylopic.org) and are available under the Public Domain Dedication 1.0 license (https://creativecommons.org/publicdomain/zero/1.0/)

We found that flight initiation distance was significantly shorter in bird species with a longer history of urbanization (Table 1; Fig. 1; Additional file 1: Table S4). Post hoc comparisons showed no significant difference in flight initiation distance between species urbanized before 1901 and those between 1901 and 1945 (mean difference = 0.017; 95% CI =  − 0.076 to 0.102; *p* = 0.718). However, escape distances were significantly shorter in species urbanized in 1901–1945 and those after 1945 (mean difference = 0.151; 95% CI = 0.011 to 0.229; *p* = 0.039) and also those before 1901 compared to those after 1945 (mean difference = 0.167; 95% CI = 0.030 to 0.300; *p* = 0.012).

**Table 1:** Results of a multi-predictor Bayesian phylogenetically informed regression model examining variation in flight initiation distance (response variable) among urban bird species in Prague (Czech Republic). The model included a categorical predictor representing the time of Prague urbanization (three-level factor: ≤ 1900—reference level; 1901–1945; ≥ 1946) and urban tolerance (i.e., breeding commonness—area of breeding occupancy in Prague), and a suite of covariates including starting distance (log10-transformed), body mass (log10-transformed), flock size (log10-transformed), time of day (hour), date, year, ambient temperature, human presence (number of people counted during each trial session), and urbanization level (core vs suburban). Species identity together with phylogenetic covariance matrix and locality were included as random effects. Parameter estimates (posterior means) are presented with their 95% credible intervals (2.5% and 97.5% CI, respectively) and Bayesian *p* values (*pMCMC*). Marginal *R*^2^ = 0.205, conditional *R*^2^ = 0.551. The analysis was based on 4420 observations from 68 bird species. Statistically significant results are highlighted in bold

Predictor	Posterior mean	2.5% CI	97.5% CI	pMCMC
(Intercept)	29.790	− 52.900	99.660	0.297
Urbanization time (1901–1945)	0.017	− 0.070	0.107	0.718
**Urbanization time (≥ 1946)**	**0.168**	**0.032**	**0.302**	**0.012**
Urban tolerance	− 0.012	− 0.045	0.022	0.459
**Starting distance**	**0.067**	**0.058**	**0.075**	** < 0.001**
Body mass	0.059	− 0.003	0.127	0.080
Flock size	0.005	− 0.003	0.013	0.220
Hour	− 0.016	− 0.033	0.002	0.081
Julian date	0.000	− 0.003	0.004	0.715
Year (continuous)	− 0.014	− 0.049	0.026	0.306
Temperature	− 0.006	− 0.017	0.003	0.234
Human presence	0.003	− 0.008	0.014	0.612
Urban level (suburban)	0.058	− 0.007	0.121	0.087

In the main model (Table 1), flight initiation distance was positively associated only with starting distance. There was no significant association between flight initiation distance and urban tolerance (measured as species’ breeding commonness in the study area) and other predictors (Table 1). We also found relatively strong phylogenetic signal in our response variable with *λ* = 0.63 (median = 0.67; 95% CrI = 0.10–0.98, Pr[*λ* > 0] = 1.00), indicating strong phylogenetic structuring of species differences in flight initiation distance. Finally, we did not find evidence for linear relationship between flight initiation distance and urbanization year (Additional file 1: Table S3).

## Discussion

Our study indicates that urbanization time, but not urban tolerance, is significantly correlated with avian fear responses towards humans (measured as escape distance) in urban populations of birds. Specifically, bird species that started to breed in Prague before 1901 exhibited significantly shorter flight initiation distances compared to species that only colonized urban habitats after the mid-twentieth century. These results are in line with the hypothesis that reduced fear responses in urban wildlife partially reflect their long-term exposure to urban environments, with earlier urbanized species having undergone stronger filtering or selection for reduced fear responses towards humans [21–24]. More broadly, birds often behave differently across the urban–rural gradient—urban populations typically show shorter escape distances and altered vigilance relative to birds from rural and natural areas—so our pattern fits this well-documented shift in risk responses under city life [3, 9, 10].

Our results suggest that differences in fear responses between long- and short-urbanized bird species are primarily shaped by long-term processes, such as behavioral filtering and positive selection for boldness [18, 19, 22], rather than short-term plastic responses like habituation. Early urban colonizers may have consisted of individuals already exhibiting low responsiveness to human presence, a trait that enabled initial persistence in the human-dominated environment [18, 22]. Although habituation-like mechanisms likely contribute to reduced responsiveness during urban colonization [15–17], they cannot fully explain the consistent variation in fear responses associated with urbanization time observed in our dataset. Species that have been breeding in Prague for extended periods exhibited persistently shorter flight initiation distances, suggesting that prolonged exposure to non-threatening human presence, relaxed predation pressure, and possibly also selective advantages of boldness and other fitness benefits of low fearfulness may have led to the stabilization of low-fear behavioral phenotypes [3, 17, 28]. If reduced fear of humans was primarily a plastic response to urban conditions, we would expect recently urbanized species to exhibit similarly short escape distances—but this was not the case.

The weaker fear responses in ancient urban colonizers compared to recent urban settlers underscore the importance of historical exposure in shaping birds’ escape behavior. The lack of significant differences in fear responses between species urbanized before 1901 and those between 1901 and 1945, and between mid-century and post-1945 colonizers, suggests that a critical threshold for detectable behavioral adjustment may require several decades of urban residence. This may indicate that some threshold reflecting the time needed for population-level processes—such as natural selection, reduced behavioral plasticity, and loss of fear variation—is necessary to take effect and become consistently expressed [4, 22, 29]. While urban–rural differences in escape behavior can arise relatively quickly following colonization of human-dominated environments [30], our findings show that initial reduction in fear—regardless of mechanisms—is further reinforced over time. As urbanization time increases, species display progressively (but perhaps not linearly, see Additional file 1: Table S3) lower fear responses towards humans, supporting the idea that long-term coexistence imposes cumulative selective pressures that result in stable, trait-based reductions in antipredator behavior.

In contrast, urban tolerance, estimated either as the spatial commonness of breeding birds within Prague or using species-specific range-wide night-time lights, did not significantly predict bird fear responses. While urban tolerance, typically estimated as intensity of night-time lights in the species distribution, has often been studied in relation to morphological and ecological adaptations of birds to anthropogenic environments [31–34], our results suggest that this measure alone may not reflect species’ behavioral adjustments in terms of their direct tolerance towards human presence. Instead, high urban tolerance may reflect cognitive skills [34, 35], ecological generalism [31, 35], life-history traits such as body size [31, 36], and reproductive and dispersal strategies [31, 35], none of which necessarily implies reduced antipredator behavior. Moreover, present urban tolerance of birds may primarily reflect recent demographic processes, such as population changes or range expansion in some species [37–39], rather than long-term behavioral adaptation to human presence. Hence, our findings support the interpretation that urbanization time—as a proxy for historical exposure of species and populations to urban environments—better captures the selective pressures shaping antipredator behavior, and more reliably explains species’ escape responses to humans than contemporary urban commonness alone.

Beyond urbanization-related variables, we found that escape distance of birds was positively associated with starting distance. The positive relationship between starting distance and flight initiation distance may reflect sensory or cognitive constraints, perceived threat persistence, or methodological artifacts, yet the exact mechanisms remain unclear [8, 40–43].

Some of our models revealed effects of other predictors such as daytime, year, urbanization level, or body size. For example, the changes in avian escape distance with daytime may reflect shifts in risk responsiveness driven by foraging urgency, with birds exhibiting reduced vigilance during early morning hours when starvation risk is highest, and increased vigilance later in the day as energy reserves are replenished [44]. Nevertheless, these effects should be interpreted very cautiously: they were included to control for potential confounding rather than to test specific hypotheses, and their effect sizes were small. No significant effects of flock size, date, temperature, or human presence (despite these being reported as predictors of escape behavior in some former studies [3, 9, 45, 46]) may result from limited variation across the studied species and relatively homogeneous sampling conditions across trials.

## Conclusions

In conclusion, our study demonstrates that urbanization time is one of key determinants of avian escape behavior in cities. Our findings contribute to the growing evidence suggesting that reduced fear of humans in urban wildlife cannot be attributed solely to current ecological conditions or recent demographic patterns, but rather reflects a deeper history of species’ coexistence with humans. This finding underscores the importance of incorporating historical dimensions of urban colonization into behavioral ecology and urban wildlife management frameworks. Moreover, by explicitly separating urbanization time from urban tolerance, we provide evidence that temporal exposure is a stronger predictor of escape behavior than present-day prevalence. This distinction is crucial for understanding the mechanisms underlying behavioral adaptation to urban environments. Future work should further explore the interaction between behavioral variation, urban colonization history, and population-level consequences such as survival, reproductive success, and long-term persistence in cities.

## Methods

### Flight initiation distance

The field data were collected during four seasons (2021–2025) in urban parks and cemeteries in the city of Prague (50.081°N, 14.426°E; 1.3 million inhabitants, 177–399 m a. s. l.), Czechia (Additional file 2: Fig. S1). Data were typically collected from well-urbanized sites (i.e., general urban zone, urban core zone, and urban center zone sensu (47)) (see also Additional file 2: Fig. S1). Birds were approached during the morning (5:00–10:00) when they are most active and during the spring period (May–June) when most sampled bird species in the study region are sedentary and territorial. All field data were collected only during favorable weather conditions (no heavy rain, mist, fog, or strong wind) and were collected by a single observer (P.M.).

Escape responses in birds are known to be highly repeatable at the individual, population, and species levels, but they are also context-dependent [7, 19, 29, 48]. To minimize potential confounding factors, we collected all data following standardized protocols and procedures described elsewhere [40, 49–51]. At each study site, a single observer (P.M.) sequentially approached as many birds as possible to estimate their escape responses. Upon detecting a focal bird, the observer began walking towards it at a normal pace (~ 1 m/s), with their head and gaze directed at the bird. Flight initiation distance (in meters) was then estimated as the distance between the observer and the bird at the moment the bird began to flee (by flying, running, or swimming). This distance was measured either by counting ~ 1-m-long steps (used in 2022–2025) or with a rangefinder (Braun Range Finder 600WH, ± 1 m; used in 2021). Validation trials confirmed that estimates from the step-count method and rangefinder were nearly identical (*N* = 20 experimental trials; Pearson’s *r* = 0.995, *t* = 42.026, df = 18, *p* < 0.001).

For birds located above ground level (e.g., perching in vegetation), escape distance was calculated as the Euclidean distance—i.e., the square root of the sum of the squared horizontal distance and squared height—when using the step method, or measured directly with the rangefinder. Only adult birds engaged in non-antipredator behaviors (mostly feeding) at the time of initial detection were approached. The observer always wore outdoor clothing in non-bright, neutral colors (typically black).

To avoid repeated sampling of the same individuals, birds were sampled at each locality only once per year. During each session, we sampled only clearly distinct conspecifics (e.g., observed simultaneously, positioned at different spots, or distinguishable based on morphological traits such as sex-specific coloration or age).

### Urbanization time, urban tolerance, and other predictors

Data on the time of urbanization were extracted from literature specifically dedicated to birds of Prague [52–54]. Authors of these sources typically collected data via their own field observations, extracted records from older sources, and/or noted observations communicated by colleagues/enthusiasts. As the time of urbanization, we considered a year when first breeding of a species was documented or probably occurred in the urban habitats of Prague. However, the exact year of bird first breeding in Prague was often not explicitly mentioned, especially for records from the nineteenth century and first half of twentieth century. Hence, we rather used a broad categorization of species into three groups based on approximate year of first breeding in Prague: 1 =  ≤ 1900 (*N* = 1749 observations for 19 species), 2 = 1901–1945 (*N* = 1189 and 35), and 3 = 1946–present (*N* = 1482 and 14). The thresholds were selected to reflect the historical development of Prague. The first category reflects pre- and early-industrial era; the second category corresponds to the period of rapid economic development and city expansion after full industrialization; the third category focuses on transformation of the city to the modern conditions after World War II. Moreover, all three categories are relatively balanced in the number of sampled observations. We excluded five species that were not reported in the above mentioned publications, as they probably still do not breed in Prague: *Aix galericulata*, *Aix sponsa*, *Anas clypeata*, *Anas penelope*, and *Dendrocopos syriacus* with only a few escape distance observations available (*N* = 1, 1, 1, 1, and 2, respectively).

We estimated the urban tolerance of birds (following definition, e.g., in [31, 32]) based on their breeding commonness within the municipality of Prague, using data from [53]. Specifically, we counted the number of grid cells in which each species had confirmed or probable breeding records. Authors of the Prague bird breeding atlas mapped the city in 1.50 × 1.25 km grid cells (total = 277) and, over one to five breeding seasons, visited all habitats in each square, recording for every species the highest breeding-evidence code under the standard European scheme together with semi-quantitative abundance classes (sensu [55]). Although the Prague municipality includes both highly and less urbanized areas, our proxy of urban tolerance should be robust. Species widely regarded as well-urbanized in Prague city occupy most atlas grid cells (e.g., *Columba livia*: 203/277, *Pica pica*: 220/277), and—to our knowledge—no species in the municipality of Prague that is common outside the city yet absent from it, aligning with the general pattern across European cities that large-scale commonness outside the cities best predicts presence in cities [56]. While previous studies have used proxies of urban tolerance such as night-time lights to estimate urban tolerance (e.g., [31, 32, 57]), we opted to quantify urban tolerance using breeding occurrence data tied to administrative grid cells within Prague’s municipal boundaries. Night-time light intensity may overestimate urbanization (e.g., due to light pollution in peri-urban or rural areas) or fail to detect fine-scale heterogeneity in habitat use within cities [58, 59]. In contrast, administrative boundaries offer a well-demarcated spatial framework that aligns with land-use planning, infrastructure, and human density, and better reflects the socio-ecological context of urban environments. We consider this an ecologically relevant indicator of urban tolerance, as the ability to persist and breed within urban-associated habitats reflects a species’ capacity to breed in anthropogenetically highly disturbed and modified areas. However, we also extracted species-specific (and range-wide) remotely sensed VIIRS night-time light values from [57] to test performance of this alternative measure of urban tolerance.

The flight initiation distance of birds is influenced by several life-history, contextual, and environmental traits [9, 10, 48] which may confound associations between escape responses of birds and urbanization time and urban tolerance, respectively. Hence, we collected information on the following variables: (1) The starting distance—the distance to the focal bird (in meters) when an observer started the escape distance trial. (2) The flock size—the number of all conspecific bird individuals moving, feeding, or perching together that were visually separated from other conspecific or mixed-species individuals. We did not approach mixed-species bird flocks. (3) The species-specific body size—estimated as the mean female and male body mass (in grams) from EltonTraits 1.0 database [60]. (4) The daytime—the time (hh:mm format) of data collection converted to hour [61]. (5) The date—date of data collection was noted as a “Julian” day since the start of the breeding season (day 1 = 1 April). (6) The year—the year of data collection was used as a categorical variable. (7) The ambient temperature—the air temperature (°C) at the site during data collection. (8) The human presence—the number of humans within a 50 m radius during each sampling session. (9) The urbanization level—we divided sites based on whether they were located in well-urbanized center and core city area (core) or in less urbanized residential and city margin areas (suburban). (10) The site—a unique identifier of each sampled park and cemetery.

### Statistical analysis

All statistical analyzes were performed in R software v. 4.1.1 [62]. We analyzed the effects of urbanization time, urban tolerance, and other contextual and environmental traits on fear responses of birds using Bayesian phylogenetic generalized linear mixed models via the *MCMCglmm* v. 2.36 package [63, 64]. We fitted a Gaussian response model with an identity link with flight initiation distance (log10-transformed) as a response variable and urbanization time (categorical), urban tolerance, starting distance (log10-transformed), body size (log10-transformed), flock size (log10-transformed), daytime (hour), date, year (continuous), temperature, human presence, and urbanization level as response variables. Before analysis, we checked collinearity between predictors using the chart. Correlation function in PerformanceAnalytics v. 2.0.4 package [65]. We generally found no serious collinearity between predictors (|*r*|≤ 0.50). All continuous predictors were centered and scaled before analysis (mean = 0, SD = 1) to facilitate interpretation [66]. We incorporated a species (controlling for non-phylogenetic species effects) and site ID as random effects. We specified uninformative inverse-gamma priors for random effects and an uninformative prior for residual variance.

To further test robustness of our results in relation to different urbanization level across sampling site, we also ran a model where we excluded data from four large parks located at the periphery of Prague (Kosikovske nadrze, site ID = 12; Hostivarsky lesopark, 14; Cimicky haj, 26; and Ďáblický háj, 23; for further details on localities, see our primary data [67]), revealing qualitatively same results regarding the effect of urbanization time (Additional file 1: Table S1). We also replaced urban tolerance based on breeding commonness by that based on night-time light values, revealing qualitatively the same results (Additional file 1: Table S2). Hence, we present only results based on all observations, categorized urbanization time and urban tolerance based on breeding commonness in the main text. Finally, we also ran a model with the original urbanization year (continuous variable) as a predictor (Additional file 1: Table S3).

We employed phylogenetically informed regression models to account for phylogenetic relationships among species while modeling escape responses of birds as a function of predictors. To control for phylogenetic structure, we first generated 100 phylogenies (using the Hackett backbone) using *BirdTree.org* online tool [68]. We used these phylogenies to reconstruct a maximum credibility tree (which returns the posterior tree with the highest clade credibility and retains its edge lengths) using maxCladeCred function in the *phangorn* v. 2.11.1 package; this provides a standard, computationally tractable summary for downstream comparative analyzes [69]. We then computed a phylogenetic covariance matrix using the *inverseA* function and included it in our models. All models were run for 300,000 iterations with a burn-in of 100,000 and a thinning interval of 100 to reduce autocorrelation. We assessed model convergence using the Gelman–Rubin statistic (running four chains) [70] to ensure that within-chain and between-chain variances were comparable. We examined posterior fixed effect distributions using 95% highest posterior density intervals to determine statistical significance. Marginal (variance explained by fixed effects only) and conditional (variance explained by the fixed and random effects) effect sizes (*R*^2^) were computed using the posterior means of the fixed and random variance components. Finally, we estimate phylogenetic signal (Pagel’s *λ*) in our response variable as the share of between-species variation that is attributable to shared ancestry, computed as the variance from the phylogenetic species effect relative to the total between-species variance (phylogenetic + non-phylogenetic), and then summarized its posterior and its probability (Pr) that *λ* > 0; values close to 1 indicate strong evidence of non-zero phylogenetic signal.

## Supplementary Information

Additional file 1: Tables S1–S4. Table S1 Model results after excluding large peripheral parks. Table S2 Model results with artificial light at night as an urban tolerance proxy. Table S3 Model results birds with continuous urbanization time. Table S4 Species-level summary of flight initiation distance.

Additional file 2: Figure S1. Fig. S1 The map of sampling sites.

## Data Availability

Primary data are available at figshare (10.6084/m9.figshare.30135715).
