# Supplementary material for: Species’ urbanization time but not present urban tolerance predicts avian fear responses towards human
Source: BMC Biol. 2025 Oct 2;23:295. doi: 10.1186/s12915-025-02427-0 (PMC12492597; doi:10.1186/s12915-025-02427-0)
Supplement: Supplementary file 1 — Additional file 1: Tables S1–S4. Table S1 Model results after excluding large peripheral parks. Table S2 Model results with artificial light at night as an urban tolerance proxy. Table S3 Model results birds with continuous urbanization time. Table S4 Species-level summary of flight initiation distance. [file 12915_2025_2427_MOESM1_ESM.pdf]

Supplementary Tables for

Species' urbanization time but not present urban tolerance predicts avian fear responses towards human

Peter Mikula*, Jan Grünwald & Jiří Reif

*✉ petomikula158@gmail.com

Figure S1. Interactive map of bird flight initiation distance (FID) observations across urban green spaces in Prague, Czechia. Each point represents a unique sampling location and point (ID.Area_Point; e.g. 47_1 means locality number 47 and point number 1 within this locality), with colour indicating site type (green = park, black = cemetery). Popups display the location name, site type, and the number of individual observations per point. **For interactive map, see Additional file 1: Fig. S1.** The lower picture shows only preview.

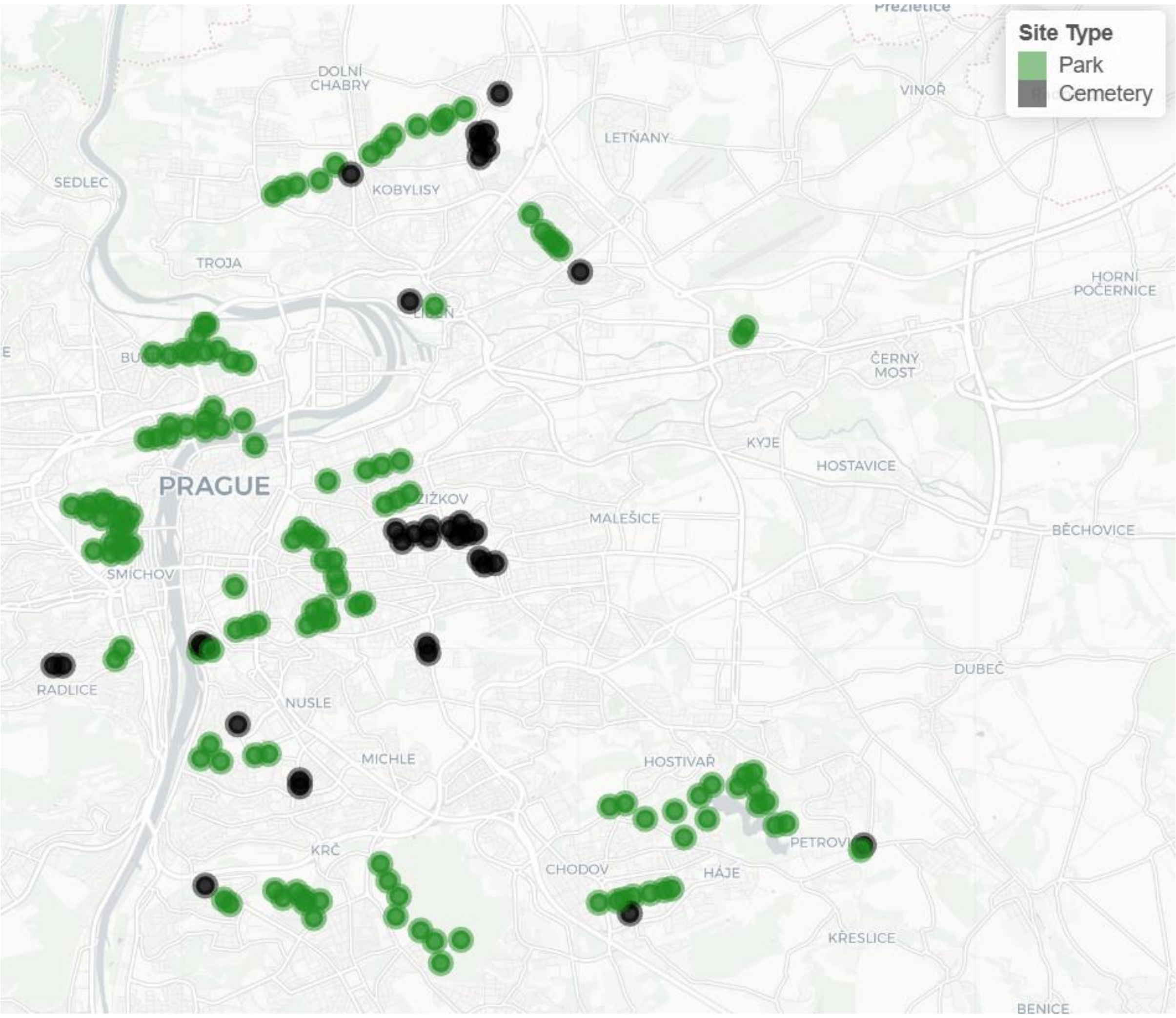

Table S1. Results of a multi-predictor Bayesian phylogenetically informed regression model examining variation in flight initiation distance (response variable) among urban bird species in Prague (Czech Republic) after excluding data from four large peripheral parks. The model included a categorical predictor representing the year of urbanization and urban tolerance (breeding commonness), and a suite of covariates including starting distance (log10-transformed), body mass (log10-transformed), flock size (log10-transformed), time of day (hour), date, year, ambient temperature, human presence (number of people counted during each trial session), and urbanization level (core vs suburban). Species identity together with phylogenetic covariance matrix and locality were included as random effects. Parameter estimates (posterior means) are presented with their 95% credible intervals (2.5% and 97.5% CI, respectively) and Bayesian *p*-values (*pMCMC*). Marginal R^2 = 0.236, conditional R^2 = 0.572. The analysis was based on 3,738 observations from 61 bird species. Statistically significant results are highlighted in bold.

**Predictor**	**Posterior mean**	**2.5% CI**	**97.5% CI**	**pMCMC**
(Intercept)	0.875	0.691	1.081	<0.001
Urbanization time (1901–1945)	0.003	-0.094	0.100	0.956
**Urbanization time (≥1946)**	**0.209**	**0.057**	**0.336**	**0.004**
Urban tolerance	-0.003	-0.034	0.031	0.877
**Starting distance**	**0.063**	**0.054**	**0.072**	**<0.001**
Body mass	0.061	-0.013	0.138	0.094
Flock size	0.006	-0.003	0.015	0.229
Hour	-0.012	-0.025	0.001	0.077
Julian date	0.001	-0.001	0.002	0.383
**Year**	**-0.021**	**-0.033**	**-0.009**	**0.001**
Temperature	-0.006	-0.016	0.005	0.285
Human presence	0.004	-0.006	0.015	0.447
Urban level (suburban)	0.055	-0.014	0.125	0.126

Table S2. Results of a multi-predictor Bayesian phylogenetically informed regression model examining variation in flight initiation distance (response variable) among urban bird species in Prague (Czech Republic). The model included a categorical predictor representing the year of urbanization and urban tolerance (night-time light), and a suite of covariates including starting distance (log10-transformed), body mass (log10-transformed), flock size (log10-transformed), time of day (hour), date, year, ambient temperature, and human presence (number of people counted during each trial session). Species identity together with phylogenetic covariance matrix and locality were included as random effects. Parameter estimates (posterior means) are presented with their 95% credible intervals (2.5% and 97.5% CI, respectively) and Bayesian *p*-values (*pMCMC*). Marginal R^2 = 0.209, conditional R^2 = 0.555. The analysis was based on 4,407 observations from 63 bird species. Statistically significant results are highlighted in bold.

Predictor	Posterior mean	2.5% CI	97.5% CI	pMCMC
(Intercept)	0.874	0.682	1.089	<0.001
Urbanization time (1901–1945)	-0.020	-0.122	0.077	0.677
**Urbanization time (≥1946)**	**0.196**	**0.054**	**0.327**	**0.004**
Artificial light at night (ALAN)	-0.047	-0.096	0.009	0.084
**Starting distance**	**0.067**	**0.059**	**0.075**	**<0.001**
Body mass	0.060	-0.010	0.124	0.078
Flock size	0.005	-0.003	0.014	0.202
**Hour**	**-0.015**	**-0.028**	**-0.003**	**0.020**
Julian date	0.000	-0.001	0.002	0.436
**Year (continuous)**	**-0.020**	**-0.032**	**-0.009**	**0.002**
Temperature	-0.006	-0.016	0.004	0.217
Human presence	0.003	-0.009	0.012	0.632
**Urban level (suburban)**	**0.060**	**0.002**	**0.120**	**0.046**

Table S3. Results of a multi-predictor Bayesian phylogenetically informed regression model examining variation in flight initiation distance (response variable) among urban bird species in Prague (Czech Republic). The model included following predictors: year of urbanization (continuous), urban tolerance (breeding commonness), and a suite of covariates including starting distance (log10-transformed), body mass (log10-transformed), flock size (log10-transformed), time of day (hour), date, year, ambient temperature, human presence (number of people counted during each trial session), and urbanization level (core vs suburban). Species identity together with phylogenetic covariance matrix and locality were included as random effects. Parameter estimates (posterior means) are presented with their 95% credible intervals (2.5% and 97.5% CI, respectively) and Bayesian *p*-values (*pMCMC*). Marginal R^2 = 0.150, conditional R^2 = 0.541. The analysis was based on 4,420 observations from 68 bird species. Statistically significant results are highlighted in bold.

Predictor	Posterior mean	2.5% CI	97.5% CI	pMCMC
(Intercept)	0.948	0.765	1.145	<0.001
Urbanization time (continuous)	0.036	-0.011	0.085	0.140
Urban tolerance	-0.016	-0.051	0.014	0.337
**Starting distance**	**0.067**	**0.059**	**0.076**	**<0.001**
**Body mass**	**0.071**	**0.005**	**0.139**	**0.042**
Flock size	0.005	-0.003	0.013	0.233
**Hour**	**-0.015**	**-0.027**	**-0.003**	**0.016**
Julian date	0.000	-0.001	0.002	0.423
**Year (continuous)**	**-0.020**	**-0.032**	**-0.009**	**<0.001**
Temperature	-0.007	-0.017	0.002	0.166
Human presence	0.003	-0.008	0.013	0.620
Urban level (suburban)	0.058	-0.001	0.120	0.061

Table S4. Summary table showing species means for flight initiation distance (FID) (m), its standard deviation (SD) (m), and sample size per each species. Table divides species based on their urbanization time (Period).

Species	N	Mean FID (m)	SD FID (m)
**≥1946**			
Accipiter nisus	2	51.5	2.12
Alopochen aegyptiaca	1	18	NA
Ardea cinerea	15	27.73	13.2
Aythya fuligula	9	16.89	1.69
Buteo buteo	4	56.05	23.36
Columba palumbus	938	10.68	7.53
Cygnus olor	5	15.4	8.2
Dendrocopos medius	5	11.11	8.62
Falco tinnunculus	3	37.77	17.96
Mergus merganser	1	39	NA
Pica pica	421	16.39	10.4
Streptopelia decaocto	53	10.85	6.23
Tachybaptus ruficollis	1	43	NA
Turdus pilaris	24	8.18	4.79
**1901–1945**			
Acrocephalus arundinaceus	1	3.61	NA
Aegithalos caudatus	34	4.62	2.03
Carduelis carduelis	11	9.62	4.75
Carduelis chloris	2	9.53	2.08
Carduelis spinus	1	13	NA
Certhia brachydactyla	49	4.93	2.43
Certhia familiaris	5	7.32	0.85
Coccothraustes coccothraustes	7	15.15	3.91
Corvus corone	6	35.31	18.52
Cuculus canorus	1	15.23	NA
Dendrocopos major	116	13.32	8.68
Dryocopus martius	7	24.53	12.54
Emberiza citrinella	2	9	4.24
Erithacus rubecula	86	8.27	5.12
Ficedula albicollis	7	9.45	2.05
Ficedula hypoleuca	2	7.67	0.55
Fulica atra	6	5.33	1.63
Garrulus glandarius	142	11.59	8.41
Oriolus oriolus	3	21.93	4.58
Parus caeruleus	93	4.86	2.36
Parus palustris	2	10.92	7.19
Passer montanus	20	7.23	3.55
Phasianus colchicus	1	15	NA
Phylloscopus collybita	62	6.69	2.97
Phylloscopus sibilatrix	16	9.68	2.56

Phylloscopus trochilus	5	6.05	2.11
Prunella modularis	1	19.21	NA
Regulus ignicapilla	2	4.3	0.99
Sitta europaea	83	4.94	2.59
Sturnus vulgaris	165	14.34	7.6
Sylvia atricapilla	150	7.06	3.26
Sylvia borin	3	7.55	1.75
Troglodytes troglodytes	33	8.81	2.95
Turdus philomelos	60	10.77	7.87
Turdus viscivorus	5	12.79	9.14
**≤1900**			
Anas platyrhynchos	98	9.52	9.5
Columba livia	307	4.1	3.87
Corvus frugilegus	12	23.36	19.58
Corvus monedula	158	9.54	7.4
Fringilla coelebs	85	7.04	3.94
Gallinula chloropus	21	14.48	6.76
Hippolais icterina	1	9.49	NA
Luscinia megarhynchos	27	4.93	1.61
Motacilla alba	4	13.55	5.95
Motacilla cinerea	7	16.46	4.81
Muscicapa striata	2	11	1.41
Parus major	312	5.28	3.14
Passer domesticus	2	8.02	4.27
Phoenicurus ochruros	26	11.05	5.45
Phoenicurus phoenicurus	93	9.96	4.22
Picus viridis	65	20.38	12.46
Sylvia communis	10	9.48	3.92
Sylvia curruca	17	5.87	1.83
Turdus merula	502	9.74	8.47
